# Calixmexitil: Calixarene-based Cluster of Mexiletine with Amplified Anti-myotonic Activity as A Novel Use-dependent Sodium Channel Blocker

**DOI:** 10.22037/ijpr.2019.1100768

**Published:** 2019

**Authors:** Amirali Delnavaz Shahr, Fazel Nasuhi Pur

**Affiliations:** a *Department of Chemistry, Faculty of Science, Urmia University, Urmia, Iran. *; b *Health Technology Incubator Center, Urmia University of Medical Sciences, Urmia, Iran.*

**Keywords:** Mexiletine, Cluster, Calixarene, Antimyotonic activity, Amplify, Sodium channel blocker

## Abstract

Mexiletine as the first choice drug in myotonia treatment is a chiral sodium channel blocker clinically used in its racemic form. The phenolic structure of this drug, prompted us to design its novel calix[4]arene-based cluster in a chalice-shaped structure. Therefore, the present study reports the synthesis and *in-vitro* anti-myotonic activity of the chalice-shaped cluster of mexiletine (namely calixmexitil) in comparison to its simple drug unit (mexitil) as the reference medication. The synthetic route included chemical modification of the calix[4]arene structure by grafting four 2-aminopropoxy moieties at the lower rim of the scaffold. Electrophysiological tests were performed for the determination of test compounds abilities to act as sodium channel blockers in inhibiting sodium currents (in use-dependent manner) in single skeletal muscle fibers. The experimental results showed an amplified (10-fold) potency in producing phasic block as an indication of the anti-myotonic activity and improved (3-fold) potency in producing use-dependent block for the cluster (calixmexitil) in relation to its monomer (mexiletine). The potency in producing phasic block and use-dependent block are two main factors to describe dose range, drug affinity, and side effects of an anti-myotonic agent. Therefore, compared to mexiletine, calixmexitil with these improved factors can be considered as a “selective” anti-myotonic agent with low dose range. These improved pharmaceutical effects are maybe attributed to clustering effect and improved interaction of four impacted mexiletine units of the cluster with the sodium channels’ structure in skeletal muscle fibers.

## Introduction

Costs on new drug discovery researches have compelled medicinal chemists to look for new strategies to obtain novel active pharmaceutical ingredient (1). One of successful and low-cost strategies is the clustering of the established therapeutic drug agents on a suitable molecular scaffold in order to improve their pharmaceutical efficiencies (2).

The clinical term myotonia (muscle tension) is a skeletal muscle stiffness and it is characterized by abnormal delayed relaxation of the skeletal muscles after electrical stimulation or contraction (3). Mexiletine (mexitil) or 1-(2,6-dimethylphenoxy)-2-propanamine is a chiral non-selective voltage-gated sodium channel blocker, that is clinically used in its racemic form as an anti-myotonic, anti-arrhythmic, and analgesic agent (3-5). Mexiletine is a class Ib anti-arrhythmic drug that is the first choice drug in myotonia treatment with use-dependent behavior in inhibiting sodium currents in skeletal muscle fibers (6). By blocking sodium channels in skeletal muscle in a use-dependent manner, mexiletine inhibits the myotonic discharges of action potentials and favors muscle relaxation (6). In the other words, anti-myotonic drugs such as mexiletine facilitate the relaxation of skeletal muscles after electrical excitation. However, mexiletine as an important anti-myotonic drug has some disadvantages such as the high-therapeutic dose range, narrow therapeutic index, and serious side effects at cardiac and central nervous system levels upon chronic treatments (7, 8). Thus, the symptomatic therapy of myotonic syndromes needs to be improved for a selective treatment and reducing therapeutic dose range by means of a novel anti-myotonic agent. This goal can be achieved by common expensive new drug discovery researches or by low-cost clustering the established anti-myotonic drug units (*i.e.*, mexiletine) on a suitable molecular scaffold as an alternative strategy for reducing the costs of researches. It should be noted that the clustering effect improves the bio-activity of the certain medication via impacting drug units in the cluster structure and consequently synergizing the pharmacological effects of the units.

On the other hand, calixarenes, phenol-formaldehyde cyclic oligomers with three-dimensional structures, are suitable structures (without any notable *in-vivo* toxicity and immune responses) for designing and developing new drugs via clustering of single phenolic drug units such as mexiletine (9-14). So, in our chained studies for the synthesis of new calixdrugs (calixarene-based clusters of established therapeutic drug agents), in the present study, a novel calix[4]arene-based cluster of mexitil with chaliced shape has been reported and evaluated for its potentially enhanced bio-activity in inhibiting sodium currents in single skeletal muscle fibers in a use-dependent manner (anti-myotonic activity) with respect to its monomer mexiletine as reference drug (2). Based on calixarenes’ nomenclature, the chalice-shaped cluster of mexitil was innovatively named calixmexitil (2). 

In summary, the main purpose of this paper is to describe the role of clustering effect, as an alternative and low-cost strategy for reducing the costs on drug discovery researches, in improvement of pharmaceutical properties and reducing side effects of mexiletine as the first choice drug in myotonia treatment via comparative study of drug affinity, use-dependent behavior, dose range of the cluster compound (calixmexitil) with regards to its monomer mexitil.

## Experimental

*Apparatuses and chemicals*

The melting points of all compounds were recorded on Philip Harris C4954718 apparatus without calibration. IR spectra were determined on a Thermo Nicolet 610 Nexus FT-IR spectrometer in KBr disks. ^1^H (300 MHz) and ^13^C (75 MHz) NMR measurements were recorded on a Bruker AVANCE spectrometer in CDCl_3_ using TMS as the internal reference. Elemental analyses were performed using a Heraeus CHN-O-Rapido analyzer. Mass spectra were recorded on a JEOL-JMS 600 (FAB MS) instrument. Thin layer chromatography (TLC) analyses were carried out on silica gel plates. All chemicals were purchased from Merck, Sigma Aldrich, and Fluka Chemie (Tehran, Iran) and used as received by standard procedures. All reactions were carried out under a nitrogen or argon atmosphere.

*Synthetic procedures*

*25,26,27,28-Tetrahydroxycalix[4]arene (1)*

A slurry of *p*-t-butylcalix[4]arene (4 g, 6 mmol), phenol (2.82 g, 30 mmol) and AlCl_3_ (4.67, 35 mmol) was stirred in toluene (50 mL) at room temperature for 1 h in an inert atmosphere. The mixture was poured into 0.2 N HCl (80 mL), the organic phase was separated, and the toluene wan evaporated. Upon the addition of MeOH a precipitate formed, which was removed by filtration to give a solid. Recrystallization from MeOH-CHCl_3_ afforded compound **1** as colorless crystals.

Yield (1.96 g, 75%), mp: 314-316 °C. ^1^H NMR (300 MHz, CDCl_3_): δ 10.19 (s, 4H, OH), 7.22 (d, J = 8 Hz, 8H, Ar-H*m*) 6.64 (t, J = 8Hz, 4H, Ar-H*p*), 3.63-3.48 (bd, 8H, ArCH_2_Ar); ^13^C NMR (75 MHz, CDCl_3_): δ 148.4 (C*i*), 129.0 (C*m*), 128.2 (C*o*), 122.2 (C*p*), 31.7 (ArCH_2_Ar).

*25, 26, 27, 28- Tetrakis (methyl carbonylmethoxy)calix [4]arene (2)*

To a stirred mixture of compound **1** (1.7 g, 4 mmol) and K_2_CO_3_ (4.35 g, 30 mmol) in acetone (50 mL) was added a solution of NaI (4.65 g, 30 mmol) and chloroacetone (2.5 mL, 30 mmol) in acetone (20 mL). The reaction mixture was heated to reflux under N_2_ atmosphere for 6 h. Then, it was cooled, filtered, and washed with fresh acetone (10 mL). After solvent evaporation, the residue was suspended in water (30 mL) at 60 °C and stirred for 2 h. Then the product was extracted into the dichloromethane (20 mL). Removal of solvent, left a pale-yellow solid which on recrystallization from acetone furnished the tetra-methyl ketone **2** as white crystals. 

Yield (1.25 g, 48%), mp: 220-222 °C. IR (KBr, ν, cm ^− 1^): 1731 (C = O). ^1^H NMR (300 MHz, CDCl_3_): δ 7.20 (d, J = 7.3 Hz, 8H, Ar-H *m*), 6.69 (t, J = 7.3 Hz, 4H, Ar-H *p*), 5.02 (d, J = 13 Hz, 4H, ArCH_2_Ar, H_ax_), 4.76 (s, 8H, ArO−CH_2_), 3.19 (d, J = 13 Hz, 4H, ArCH_2_Ar, H_eq_), 2.21 (s, 12H, CH_3_); ^13^C NMR (75 MHz, CDCl_3_): δ 205.2 (C=O), 153.1 (C*i*), 136.2 (C*o*), 127.8 (C*m*), 119.2 (C*p*), 76.4 (ArOCH_2_), 31.7 (Me), 31.4 (ArCH_2_Ar). Anal. Calcd for C_40_H_40_O_8_: C, 74.06; H, 6.22. Found: C, 74.08; H, 6.19. FAB^+^MS m/z = 648.23 (M ^+^).

**Table 1:** Values of IC50 and their ratio for skeletal muscle fibers by test compounds

**Compound**	**Tonic block IC** **50 ** **(μM)**	**Phasic block IC** **50 ** **(μM)**	**Tonic/Phasic ratio**
Mexiletine	89	31	3
Calixmexitil	28	3	9

**Scheme 1:**
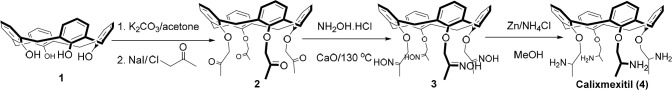
Synthetic pathway to calixmexitil

**Figure 1:**
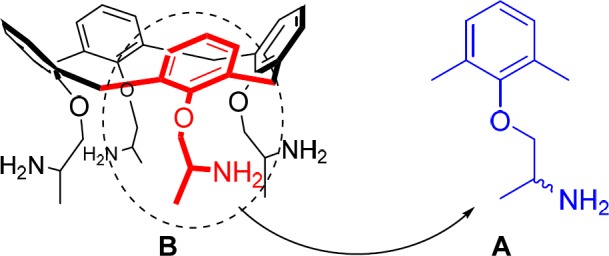
Structural comparison of (A) mexiletine as the monomer of (B) calixmexitil

*25, 26, 27, 28- Tetrakis(methyl hydroxyiminomethoxy)calix [4]arene (3)*

A mixture of compound **2** (1 g, 1.54 mmol) and calcium oxide (8.4 g, 0.15 mol) were heated to 130 °C in an oil bath for a few minutes. Then, hydroxylamine hydrochloride (3.12 g, 45 mmol) was added and the mixture was stirred with a magnetic stirrer in the presence of air for an hour. Then, the reaction mixture was mixed with ethyl acetate (50 mL), filtrate to remove CaO and mixed with water (30 mL) and extracted. The ethyl acetate solution was dried over Na_2_SO_4_. The solution was concentrated in vacuum and treated with dilute acetic acid (20 mL). The resulting yellow powder was filtered, washed with water, recrystallized from methanol and then from CH_3_OH/CHCl_3_ to give tetra oxime **3** as orange powder.

Yield (0.46 g, 42%), mp: 232-233 °C. IR (KBr, ν, cm ^− 1^): 3572 (O-H), 1629 (C = N). ^1^H NMR (300 MHz, CDCl_3_): δ 12.48 (s, 4H, N-OH), 7.32 (d, J = 7.6 Hz, 8H, Ar-H *m*), 6.77 (t, J = 7.6 Hz, 4H, Ar-H *p*), 4.89 (d, J = 13.3 Hz, 4H, ArCH_2_Ar, H_ax_), 4.69 (s, 8H, ArO−CH_2_), 3.23 (d, J = 13.3 Hz, 4H, ArCH_2_Ar, H_eq_), 2.28 (s, 12H, CH_3_); ^13^C NMR (75 MHz, CDCl_3_): δ 160.9 (C=N), 152.8 (C*i*), 138.2 (C*o*), 127.4 (C*m*), 119.9 (C*p*), 77.3 (ArOCH_2_), 32.1 (Me), 31.2 (ArCH_2_Ar). Anal. Calcd for C_40_H_44_N_4_O_8_: C, 67.78; H, 6.26; N, 7.90. Found: C, 67.76; H, 6.27; N, 7.93. FAB^+^MS m/z = 708.28 (M ^+^).

*Calixmexitil (4)*

To a mixture of compound **3** (0.35 g, 0.5 mmol) in methanol (20 mL) was added NH_4_Cl (0.8 g, 15 mmol) and zinc powder (0.64g, 10 mmol). The mixture was stirred under reflux for 12 h. Then the reaction mixture was filtered and the filtrate was evaporated under vacuum and the residue was taken into chloroform (20 mL), washed with saturated NaCl solution (10 mL) and finally with water (10 mL). The organic layer was dried over anhydrous Na_2_SO_4_ and evaporation of the organic layer was followed by recrystallization of the residue in methanol/chloroform, to yield the final product as white crystals.

Yield (0.25 g, 77%), mp: 199-201 °C. IR (KBr, ν, cm ^− 1^): 3306 (N-H). ^1^H NMR (300 MHz, CDCl_3_): δ 10.63 (bs, 8H, NH_2_), 7.48 (d, J = 7.3 Hz, 8H, Ar-H *m*), 6.84 (t, J = 7.3 Hz, 4H, Ar-H *p*), 4.96 (d, J = 13.7 Hz, 4H, ArCH_2_Ar, H_ax_), 4.53 (s, 8H, ArO−CH_2_), 3.44 (d, J = 13.7 Hz, 4H, ArCH_2_Ar, H_eq_), 2.32 (s, 12H, CH_3_); ^13^C NMR (75 MHz, CDCl_3_): δ 151.3 (C*i*), 139.0 (C*o*), 126.8 (C*m*), 118.7 (C*p*), 76.8 (ArOCH_2_), 49.2 (C-N), 31.9 (Me), 31.3 (ArCH_2_Ar). Anal. Calcd for C_40_H_52_N_4_O_4_: C, 73.59; H, 8.03; N, 8.58. Found: C, 73.62; H, 8.01; N, 8.55. FAB^+^MS m/z = 652.44 (M ^+^).

*Biological Evaluations*

*Preparation and Solutions*

For sodium current recordings, semitendinosus muscle fibers were perfused with the following “external” solution (mM): NaCl 77, choline-Cl 38, CaCl_2_ 1.8, Na_2_HPO_4_ 2.15, NaH_2_PO_4_ 0.85; and dialyzed with the following “internal” solution (mM): CsF 105, MOPS 5, MgSO_4_ 2, EGTA 5, Na_2_ATP 0.55 (pH) 7.2 with NaOH concentrated solution). Stock solutions of mexiletine and calixmexitil were prepared in physiological and/or “external” solutions. The final concentrations to be tested *in-vitro* were obtained by further diluting the stock solution as needed. DMSO at the highest concentration used (0.2%) was without effect on any of the parameters recorded.

*Voltage Clamp Recordings of Sodium Current and Pulse Protocols*

The voltage clamp recordings of sodium current were performed on single muscle fibers obtained with microsurgery from the ventral brunch of semitendinosus muscle of frog by means of the three vaseline gap voltage clamp technique as detailed elsewhere (15). After an equilibration time of 10 min, Na^+^ currents recordings were performed at 10 °C. The holding potential (hp) was -100 mV. The inward sodium traces were recorded using a voltage clamp amplifier based on that described by Hille and Campbell (16). The currents flowing in response to depolarizing command voltages were low pass filtered at 10 kHz visualized on an oscilloscope and sampled at 20 kHz. Maximal sodium currents were elicited with test pulses from the hp to -20 mV for 10 msec. Tonic block exerted by the test compounds was evaluated as percent reduction of the peak sodium current elicited by single test pulses. The evaluation of use-dependent block by the drugs was made by using a 10 Hz train of test pulses for a period of 30 sec and by normalizing the residual current at the end of this stimulation protocol with respect to that in the absence of drug.

## Results and Discussion

Due to non-selective treatment of myotonia by high dose range of mexiletine and consequently, its serious side effects, it is necessary to improve the treatment by a novel anti-myotonic agent with amplified potency, low dose range, and enhanced use-dependent behavior (7, 8). Hypothetically, it can be possible via clustering the anti-myotonic drug mexiletine units on a suitable molecular backbone. On the other hand, due to the *para*-substituted phenolic structure of mexiletine, and considering the fact that calixarenes are the cyclic oligomer of phenols, the calix[4]arene as a suitable scaffold was chosen for clustering four impacted simple phenolic mexiletine units, in a chalice-shaped structure (calixmexitil), in order to improve both drug affinity and use-dependent behavior via clustering effect (2).

The synthetic route to calixmexitil **4** is depicted in Scheme 1. The synthetic route is similar (steps and reagents) to the synthetic pathway of the parent drug mexiletine. The synthetic strategy involves the grafting of four 2-aminopropoxy moieties on the lower rim of the calix[4]arene scaffold via reduction of the related tetra-oxime calix[4]arene **3** in the presence of zinc powder and ammonium chloride in methanol under reflux condition for 12 h (conversion of oxime to amine). The compound **3** was synthetized by a solvent-free reaction of tetra-methyl ketone calix[4]arene **2** with NH_2_OH.HCl salt in the presence of calcium oxide at 130 °C for an hour (conversion of carbonyl to oxime). The compound **2** was synthetized by the reaction of calix[4]arene **1** with chloroacetone in the presence of sodium iodide and potassium carbonate in acetone under reflux condition for 6 hours. All compounds’ structures were well characterized by NMR, FT-IR, and FAB-MS spectra and elemental analyses. Due to the zone of carbons’ chemical shifts (~ 31 ppm, ^13^C NMR) of methylene bridges in the structures of calixmexitil and its parent compounds **2** and **3**, it can be concluded that the mentioned compounds have cone conformation (17).

In order to evaluate the potentially amplified biological activity of calixmexitil, it has been compared with its monomer (mexiletine) as reference drug in inhibiting sodium currents in single skeletal muscle fibers (Figure 1). For this purpose, they were evaluated for *in-vitro* activity as sodium channel blockers on single muscle fibers in a use-dependent manner.

The class IB anti-arrhythmic group of drugs (*i.e.*, mexiletine) mainly act by preventing fast inward sodium channels from opening (sodium current inhibitor). However, this block has two components: The tonic block is time-insensitive and concentration-dependent (block of sodium channel at resting conditions evaluated during infrequent depolarizing pulses), whereas the phasic block (also called use-dependent, cumulative sodium current reduction by the drug at high stimulation frequency) depends on the rate of the depolarization. Phasic block (sodium current inhibition in use-dependent manner) can be considered as an indication of the anti-myotonic activity and it occurs in condition of high frequency of stimulation (18). 

Concentrations of test channel blocker agents for half-maximal tonic and phasic block of sodium currents (IC_50_) in single skeletal muscle fiber are reported in Table 1. 

Putative anti-myotonic activity of the test compound is the potency in phasic block producing, being indicated by the value of the phasic block IC_50_. Also, a test compound with much less value of phasic block IC_50 _is more potent than the rest of other test compounds in anti-myotonic activity. 

As shown in the Table 1, calixmexitil (IC_50_ = 3 μM) is about 10-fold more potent than mexiletine (IC_50_ = 31 μM) in condition of high frequency of stimulation ([IC_50_ (mex phasic block)/IC_50_ (calix phasic block)] = 31/3 ~ 10) and consequently, it is more potent than mexiletine in considering as an anti-myotonic agent. 

On the other hand, the ratio of IC_50_ (tonic block)/IC_50_ (phasic block) for a blocking agent indicates its use-dependency manner and this behavior would be better when the value of the ratio is high (18). As shown in the Table 1, calixmexitil is 3-fold more potent than the mexiletine (9/3 = 3) in the production of tonic/phasic blocks and consequently, it was more potent than mexiletine in exerting a use-dependent block. 

In simple terms, the best anti-myotonic agent should be more potent in producing phasic block and use-dependent block in relation to the other test compounds.

Compared to mexitil, calixmexitil with less IC_50_ value for producing phasic block of sodium currents and more potency in exerting a use-dependent block is a “selective” anti-myotonic agent with “low-therapeutic dose range” and consequently, as mentioned in the introduction section (7, 8), hypothetically, via fewer side effects, it can be safer than mexiletine.

The enhanced pharmaceutical properties of calixmexitil are maybe attributed to clustering effect of four impacted mexiletine units and improved interaction of these units with the structure of the sodium channels in single muscle fibers.

In summary, the present work describes a low-cost drug discovery research for the synthesis of the first calixarene-based cluster (cyclic tetramer in chaliced shape) of mexiletine as a novel use-dependent sodium channel blocker with improved (10-fold) anti-myotonic activity and improved (3-fold) use-dependency behavior in comparison to its monomer, mexiletine as reference anti-myotonic drug. Compared to mexiletine, calixmexitil with improved potency in producing phasic block and use-dependent block is more “selective” anti-myotonic agent than mexitil in addition to possessing low-therapeutic dose range.
